# Velocity-based resistance training in institutionalized older adults: protocol for an experimental study with follow-up

**DOI:** 10.3389/fragi.2026.1827269

**Published:** 2026-05-18

**Authors:** Ángel Buendía-Romero, Héctor Gutiérrez-Reguero, Lucia Romero-Valia, Enrique Higueras-Liébana, Mikel Garcia-Aguirre, David Garcia-Albin, Álvaro Rosado Artalejo, Antonio Torres Bertos, Francisco J. Garcia-Garcia, Luis M. Alegre, Ignacio Ara, Julian Alcazar

**Affiliations:** 1 GENUD Toledo Research Group, Faculty of Sport Sciences, University of Castilla-La Mancha, Toledo, Spain; 2 Grupo Mixto de Fragilidad y Envejecimiento Exitoso UCLM-SESCAM, Universidad de Castilla-La Mancha-Servicio de Salud de Castilla-La Mancha, Instituto de Investigación Sanitaria de Castilla-La Mancha (IDISCAM), Toledo, Spain; 3 Centro de Investigación Biomédica en Red Fragilidad y Envejecimiento Saludable (CIBERFES), Instituto de Salud Carlos III, Madrid, Spain; 4 Residencia para Personas Mayores Barber de Toledo, Junta de Comunidades de Castilla-La Mancha (JCCM), Toledo, Spain; 5 Residencia para Personas Mayores el Greco, Grupo ENOC, Toledo, Spain; 6 Geriatrics Department, Hospital Universitario de Toledo, Toledo, Spain

**Keywords:** aging, exercise training, lower-limb power, nursing home, physical activity, sarcopenia

## Abstract

**Background:**

The effects of velocity-based resistance training (VBRT) in institutionalized older adults remain poorly explored. This protocol aims to describe a study designed to i) analyze the effects of a 9-month VBRT program on skeletal muscle size, muscle function, physical performance, disability, cognitive function and frailty in nursing home residents, ii) compare the impact of different velocity loss (VL) thresholds (10% vs. 20%), and iii) determine the residual effect of the intervention after 6 months exercise cessation.

**Methods:**

Nursing home residents aged ≥65 years will participate in this experimental study. Participants will complete a 4-week control period followed by a 9-month VBRT program and 6 months of follow-up after exercise cessation. The intervention will consist of VBRT twice a week using the leg press exercise for 9 months. The participants will be randomly assigned to two VL groups: 10% vs. 20% VL, performing *n* repetitions at 60% of estimated maximal isometric force until reaching their prescribed VL. To minimize training volume differences, the 10% VL group will complete 4 sets, while the 20% VL group will complete 3 sets. Outcomes will include muscle size of rectus femoris and vastus lateralis (ultrasound), muscle function (sit-to-stand muscle power and force-velocity relationship parameters), physical performance (Short Physical Performance Battery and walking tests), disability (Barthel and Lawton Indexes), cognitive function (Mini-Mental State Examination) and frailty (Fried Phenotype and Frailty Trait Scale). The time course of the exercise-induced adaptations will be assessed throughout the study.

**Discussion:**

We hypothesize that VBRT will exhibit meaningful benefits in muscle size, muscle function, physical performance, disability, cognitive function and frailty among institutionalized older adults, with no significant differences between 10% and 20% VL thresholds. While deconditioning is predictable after 6 months of exercise cessation, the investigated outcomes are expected to remain significantly and positively elevated compared to baseline values. This study will help to identify innovative actions to improve overall health and endorse exercise practice in older adults living in nursing homes.

**Trial Registration:**

ClinicalTrials.gov, identifier NCT07027397.

## 1 Introduction

Aging is inevitably accompanied by a physiological decline in endocrine, mitochondrial, neural and muscle functions (Cruz-Jentoft et al., 2010; Clark, 2019; Ara et al., 2025). These age-related alterations in conjunction with physical inactivity lead to physical dysfunction, cognitive impairment and disability in activities of daily living (ADL). All these negative consequences of aging can ultimately lead to nursing home admission (Luppa et al., 2010). Evidence suggests that institutionalized older adults constitute a vulnerable population characterized by relatively high prevalence rates of frailty and cognitive impairment (Kojima, 2015; Chen et al., 2023). These clinical conditions combined with an expected 2.2-fold increase in institutionalized older people in the next decades (Pickard et al., 2007) pose a major public health challenge. In this regard, cost-effective strategies are needed in long-term care facilities to reduce the high economic costs related to healthcare in institutionalized older adults (Tang et al., 2022).

The implementation of physical exercise programs is one of the most effective interventions for improving health and wellbeing of institutionalized adults (Valenzuela et al., 2023; Xu et al., 2023; Izquierdo et al., 2025). In particular, resistance training (RT) is widely recognized as a cornerstone intervention to mitigate, and even counteract, age-related declines in muscle mass and neuromuscular function, thereby reducing the risk of sarcopenia, frailty and disability in older adults (Fragala et al., 2019). In addition to neuromuscular adaptations, RT exerts positive effects on cognitive function (Cadore et al., 2014b; Zhu et al., 2025), mitigating age-related cognitive decline in memory, attention, and executive function of older adults.

Technological advances have provided the implementation of device-assisted approaches in RT to facilitate its monitoring and prescription, providing more tailored and potentially more effective RT interventions in younger populations (Courel-Ibáñez et al., 2019; Pérez-Castilla et al., 2019). In this regard, velocity-based resistance training (VBRT), which consist in monitoring repetition velocity and prescribing RT accordingly, is well established in healthy and athletic populations (Pareja-Blanco et al., 2020) and its use is expanding in individuals with pathologies and physical dysfunction (Jimeno-Almazán et al., 2023). VBRT not only helps to objectively determine RT intensity due to the strong relationship between concentric movement velocity and relative intensity (González-Badillo and Sánchez-Medina, 2010; Hernández-Belmonte et al., 2023a), but also RT repetition volume can be prescribed in order to achieve a desirable level of fatigue using velocity loss (VL) thresholds (Sánchez-Medina and González-Badillo, 2011). Despite the benefits of VBRT for exercise monitoring and dosing, its implementation in RT interventions in older adults is poorly explored, specifically using different VL thresholds. In this regard, previous studies with older adults recruited from community-dwelling centers have performed VBRT with 10% (Marques et al., 2022) and 20% VL (Marques et al., 2020); and only one previous study implementing a 10-week VBRT intervention with different VL thresholds has been conducted in older adults from residential care facilities or day centers (Marques et al., 2024). Although some studies have monitored velocity and VL in older adults, none have applied VBRT thresholds exclusively in institutionalized older adults, especially with different VL thresholds. Indeed, exploring different VL thresholds in this population could provide information on which stimulus is most efficient for inducing adaptations while minimizing systemic fatigue.

While traditional RT investigations in older people maximize physiological adaptations through continuous, rigid periodicity and control over short periods of time (e.g., 10 weeks), pragmatic interventions (i.e., adapted to the individuals’ routines and calendar) but longer in time (e.g., >6 months) are needed to elucidate the real-world effectiveness of exercise interventions during aging. Moreover, understanding whether VBRT can induce residual beneficial effects after exercise program cessation (Buendía-Romero et al., 2025) is crucial for designing cost-effective exercise programs in nursing homes with long-term benefits.

Against this background, the present protocol aims to describe a study designed to i) analyze the effects of one season (from Autumn to Summer; 9 months) of VBRT on skeletal muscle size, muscle function, physical performance, disability, cognitive function and frailty in older adults living in nursing homes (primary objective); ii) compare the impact of different VL thresholds (10% vs. 20% VL) (secondary objective I); and iii) determine the residual effect of the VBRT program after 6 months of exercise cessation (secondary objective II).

## 2 Materials and methods

### 2.1 Design and participants

This study protocol was registered in ClinicalTrials.gov (ID: NCT07027397) and approved by the Clinical Research Ethics Committee of the Toledo Hospital Complex (ID: 710P). The present protocol will be conducted in accordance with the SPIRIT (Standard Protocol Items: Recommendations for Interventional Trials) statement (Chan et al., 2013; Supplementary Table S1). Adults aged ≥65 years living in two nursing homes (Toledo, Spain) will participate in this research. Participants will be informed about the characteristics of the study and will provide their signed consent. The study will include a 4-week control period, followed by 9 months of VBRT in the leg press exercise and a 6-month follow-up in which no exercise intervention will be conducted. All participants will be allocated into the intervention group due to ethical reasons (Izquierdo et al., 2025), and thus the control period will serve as the comparator condition. Participants will be randomized in two experimental groups: 10%VL group or 20%VL group. A randomization sequence stratified by theoretical maximal isometric force (F_0_) and sex will be used to assign participants to both groups.

**FIGURE 1:**
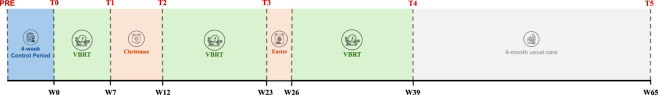
Experimental design overview. The study comprises three phases: a 4-week control period, a 9-month velocity-based resistance training (VBRT) intervention and a 6-month follow-up period. Assessment time points are indicated at specific weeks (w): T0 indicates baseline; T1-T4, intervention assessments; and T5, final follow-up.

Following our intentionally pragmatic and translational perspective to investigate the effects of VBRT on skeletal muscle size, muscle function, physical performance, disability, cognitive function, and frailty, the exercise intervention will be conducted over a whole season, including periods in which the intervention will be paused (e.g., Christmas, Easter, summer holidays) and then restarted. Thus, the main outcomes will be assessed at six specific time points as detailed in Figure 1: prior to a 4-week control period (Pre-control), at baseline (T0), post 6 weeks (T1), post 12 weeks (T2), post 23 weeks (T3), and post 39 weeks (T4). Upon completion of the VBRT, a final follow-up will be conducted after 6 months of usual care (T5).

The required sample size was determined on the basis of muscle function, using the 5-rep sit-to-stand (STS) muscle power test (Alcazar et al., 2018). According to previous research conducting exercise interventions in nursing home residents (Gutiérrez-Reguero et al., 2024), a within-group difference of +0.64 W·kg^−1^ (95%CI: +0.39 to +0.89) in relative muscle power was expected. Using this effect size (ES = 0.91), a required sample size of 15 participants was calculated in the G*Power software, assuming α = 0.05 and power = 90%. Assuming a maximum loss to follow-up of ∼35%, we will recruit 27 nursing home residents aged ≥65 years to address our primary objective. Exclusion criteria will include: i) uncontrolled hypertension, unstable or exercise-induced angina pectoris or myocardial ischemia or any other medical condition incompatible with physical exercise, ii) bedridden or unable to walk with assistance, and iii) terminal illness. The study will be performed in accordance with the Helsinki Declaration.

### 2.2 Outcomes assessment

All the outcomes will be assessed across three separate sessions, with a minimal interval of 48 h between each. On the first day, anthropometric measures and muscle size will be evaluated. The second day will focus on questionnaires and battery tests assessing disability, physical performance, frailty and muscle function. Finally, on the last day, questionnaires on cognitive function and the individual force-velocity (F-V) relationship on the leg press will be assessed. An overview of the outcomes that will be evaluated during this study is presented in Table 1.

**TABLE 1:** Overview of the outcomes.

Outcome	Device/Procedure
Anthropometry and body composition	
Body mass, *kg*	Scale
Height, *m*	Stadiometer
Body mass index, *kg·m* ^*−2*^	​
Muscle size	Ultrasound
Anatomical cross-sectional area of the rectus femoris, *cm* ^*2*^	​
Anatomical cross-sectional area of the vastus lateralis, *cm* ^*2*^ Muscle volume of the vastus lateralis, *cm* ^*3*^	​
Sit-to-stand (STS) muscle power	Power frail app
Absolute muscle power, *W*	​
Relative muscle power, *W·kg* ^*−1*^	​
Allometric muscle power, *W·m* ^*−2*^	​
Force-velocity parameters	Leg press and linear position transducer
Theoretical maximal isometric force, *N*	​
Theoretical maximal unloaded velocity, *m·s* ^*-1*^	​
Maximal muscle power, *W*	​
Slope of the F-V relationship, *N·(m·s* ^*−1*^ *)* ^*−1*^	​
Physical performance	​
Short physical performance battery, *score*	Stopwatch
10-M walking, *m·s* ^*-1*^	Stopwatch
Timed up and go, *s*	Stopwatch
30-s STS, *rep*	Power frail app
10-s STS, *rep*	Power frail app
Disability in basic and instrumental activities of daily living	
Barthel index	Questionnaire
Lawton index	Questionnaire
Cognitive function	​
Mini-mental state examination, *score*	Questionnaire
Frailty	See text for details
Fried frailty phenotype, *criteria*	​
Frailty trait scale short form, *score*	​

Abbreviation: F-V, force-velocity, STS, sit-to-stand.

#### 2.2.1 Anthropometric measures and muscle size

Anthropometric measures will include body mass, height, and body mass index (BMI) using a portable scale (Rowenta BS1500, Erbach, Germany) and stadiometer (Seca 213, Hamburg, Germany).

The anatomical cross sectional area (ACSA) of the vastus lateralis (VLa) and rectus femoris (RF) muscles will be assessed using a B-mode ultrasonography (GE Healthcare, LOGIQ F6, Chicago, Illinois) with a linear-array probe (50-mm field of view, 12 MHz). Resting ultrasound images will be collected with the participants in the supine position with their knees over a foam roller resting slightly flexed at 150° (180° = full knee extension) (Pareja-Blanco et al., 2020). After 15 min in the described position, VLa and RF ACSA will be measured at 35%, 50%, and 65% of the femur distance between the superior border of the greater trochanter (0% of the distance) and the inferior border of the lateral condyle (100% of the distance) of the right leg (Pareja-Blanco et al., 2020). These thigh lengths were found to be valid and highly repeatable to measure quadriceps femoris ACSA (Hernández-Belmonte et al., 2022b). Adhesive gaskets will be fixed to the skin at the predetermined site along the transverse measurement path to facilitate gel application and ensure a rectilinear trajectory. A panoramic image will be acquired by an experienced researcher (>200 h of ultrasonography practice), keeping the probe perpendicular to the surface of the skin and applying a minimum pressure not to compress the tissues (Hernández-Belmonte et al., 2022a). The location of the measurement points will be confirmed by documenting the probe positions on transparent sheets (Alegre et al., 2015).

The muscle ACSA will be measured by manually tracing the aponeurosis of the RF and VLa muscles (ImageJ v1.53a; National Institutes of Health, Bethesda, MD). The average ACSA value (in cm^2^) obtained from two images will be considered for further analysis (note: a third image will be analyzed when the coefficient of variation is higher than 5% (Pareja-Blanco et al., 2020; Hernández-Belmonte et al., 2023b). Additionally, muscle volume of the VLa muscle will be estimated from the VLa ACSA obtained at 35%, 50% and 65% of the femur length and insertion points of the VLa muscle in the femur (5% and 80% of the femur length) (Hogrel et al., 2015). This approach has been previously validated by Cornejo-Daza et al. (2024).

#### 2.2.2 Sit-to-stand muscle power

Muscle function will be assessed by the STS muscle power test (Alcazar et al., 2018). Mean power produced by the participants during the 5-repetition, 10-s and 30-s STS tests will be calculated using a validated equation incorporated into the “Power Frail” smartphone app (Alcazar et al., 2018):

$$Absolute\;muscle\;power\;\left(W\right)=\left(Body\;mass\cdot 0.9\cdot g\right)\cdot \frac{\left(\left[Body\;height\cdot 0.5\right]-Chair\;height\right)}{\left(\frac{\;STS\;time}{number\;STS\;reps}\right)\cdot 0.5}$$

Relative muscle power (i.e., normalized to body mass) and allometric muscle power (i.e., normalized to body size) will be calculated as follows:

Relative muscle power (W·kg)−1=Absolute muscle powerBody mass

Allometric muscle power (W·m)−2=Absolute muscle powerBody height squared

#### 2.2.3 Force-velocity relationship in the bilateral leg press exercise

The individual F-V relationship will be evaluated on a horizontal leg press (Selection MD, Technogym, Italy) using a linear position transducer (Chronojump, BoscoSystem, Barcelona, Spain) coupled to the machine (Sousa et al., 2024). The standardized procedure to assess the F-V relationship in older adults has been published elsewhere (Alcazar et al., 2017).

First, two familiarization sessions will be carried out to ensure the participants are able to perform the correct exercise technique. Additionally, the starting position on the leg press machine will be individually identified to reproduce the same range of movement in all assessments and exercise sessions. All participants will be with their feet placed on the leg press feet platform, back in contact with the seat, knee and hip joints with a comfortable angle of 90°–110° (180° = full extension), and both hands gripping the side handles.

**FIGURE 2:**
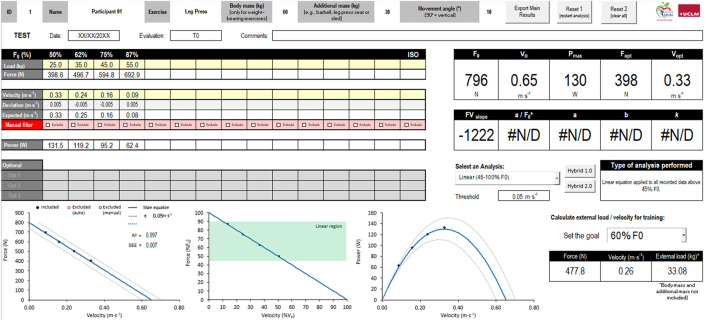
Excel-template to create the individual force–velocity (F–V) relationship in the bilateral leg press from linear position transducer data. The Excel files and instructions are publicly available at: https://github.com/JulianAlcazarPhD/F-V-profiling.

Before testing, the participants will perform a standardized warm-up including 5-min of walking at comfortable speed and 1 set of 8 repetitions on the leg press with a load equivalent to 40% of their body mass. The intended velocity will be progressively increased until conducting the last 3 reps at maximal intentional velocity. The F-V relationship will be assessed through a progressive test with at least three incremental loads until reaching 75–85%F0. The loads that did not comply with physiological principles will be discarded (Alcazar et al., 2023). The recovery time between sets will be imposed according to the mean velocity exerted by the subjects in the preceding repetition (>0.30 m·s^− 1^: 60 s; 0.30–0.20 m·s−^1:^ 90 s; <0.20 m·s^− 1^: 120 s). The subjects will be continually encouraged to perform each repetition as fast and strongly as possible. The highest mean velocity attained with each load will be considered for the analysis. A linear F-V equation will be fitted over F-V data comprised between 45% and 100% of F_0_ (Alcazar et al., 2021b). Several outcomes will be extracted from the F-V relationship (Alcazar et al., 2017): F_0_, maximal unloaded velocity (V_0_), slope of the F-V relationship, and maximal muscle power (Pmax). The F-V relationship analysis and the resulting variables are illustrated in Figure 2.

#### 2.2.4 Physical performance

The Short Physical Performance Battery (SPPB) (Guralnik et al., 1994) will be used to evaluate lower-limb physical function using three components: static balance, 4-m habitual gait speed, and 5 repetitions STS performance. For static balance, participants are instructed to stand in three different positions (feet together, semi-tandem, and full tandem) for a maximum of 10 s each. To assess gait speed, participants begin from a static position behind a line marked on the floor and walk at their usual pace toward a target located 4 m away; the test ends when they cross the target line. The time taken to complete the distance is recorded, and gait speed is calculated accordingly (m·s^-1^). For the STS test, participants start seated on a chair and perform 5 STS repetitions as quickly as possible without arms assistance. Each repetition requires contact with the seat prior to standing up again. Based on the results in each component, this battery is scored from 0 (worst) to 12 (best) with a maximum of 4 points assigned to each domain: balance, gait speed, and STS performance (Guralnik et al., 1994).

The 10-m walking test (Rossier and Wade, 2001) will be used to assess the time required to walk 10 m at maximum speed. The subjects start from a static position and walk in a straight line to complete the established distance in the shortest time possible. Maximal gait speed is calculated by dividing the distance by the time (m·s^-1^).

The Timed Up and Go test (Herman et al., 2011) will measure the time it takes the participants to (i) stand up without using their arms, (ii) walk 3 m, and (iii) turn around and sit down again. This test has been proposed for measuring agility (i.e., dynamic balance) in institutionalized older adults (Buendia-Romero et al., 2023) and as a strong predictor of fall risk among older adults (Lusardi et al., 2017).

In addition, the participants will complete the 10-s and 30- STS tests. Participants will start seated on a chair and will perform as many full STS repetitions as possible within the allotted time without arms assistance (Alcazar et al., 2020).

All tests will begin with the standardized cue “3, 2, 1, go”. Two attempts, with a rest of 1–3 min, will be allowed for each test, and the best performance will be considered for analysis. A stopwatch will be used to record the time required for timed tests and to control the duration of time-limited protocols.

#### 2.2.5 Disability in basic and instrumental activities of daily living

Disability in basic and instrumental ADL will be assessed using the Barthel index (Mahoney and Barthel, 1965) and Lawton index (Lawton and Brody, 1969). The Lawton questionnaire assesses the ability to use the telephone, shopping, cooking, household duties, laundry, use of transportation, responsibility for their medication, and handling of economic matters. Each item is assigned a numerical value (1 = independent, 0 = dependent), therefore, the maximum dependence would be marked by obtaining 0 points and total independence by obtaining 8 points. The Barthel index is a 10-item questionnaire that provides information about the autonomy to cook food, wash, dress, groom, perform bowel movements and urination, go to the toilet, move from the bed to the chair, walk and climb stairs. The total score ranges from 0 to 100, with higher scores indicating greater independence and lower scores indicating higher levels of dependence in basic daily activities.

#### 2.2.6 Cognitive function

Cognitive function will be assessed using the Mini-Mental State Examination (MMSE) questionnaire (Folstein et al., 1975). The MMSE comprises 11 items that measure attention and orientation, memory, registration, recall, calculation, language, and the ability to draw a complex polygon. The total score ranging from 1 (lowest cognitive function) to 30 (highest cognitive function) points.

#### 2.2.7 Frailty

The Frailty Trait Scale short form (FTS-5) (García-García et al., 2020) and Fried’s Frailty Phenotype (FP) (Fried et al., 2001) will be used to assess frailty syndrome. FTS-5 evaluates five domains: BMI, Physical Activity Scale for the Elderly (PASE) questionnaire, handgrip strength, Romberg static balance test and gait speed in the 3m walking test. Each FTS-5 component is scored from 0 to 10, yielding a total score ranging from 0 to 50. Participants scoring ≤25 points will be classified as robust, whereas those scoring >25 points will be classified as frail (García-García et al., 2020). FP includes 5 criteria: involuntary loss of ≥4.5 kg in the prior year, self-reported exhaustion, weakness (i.e., low handgrip strength), low habitual gait speed in the 4.5-m walking test and low physical activity measured through the PASE questionnaire. Participants will be classified as robust (0 criteria), pre-frail (1 – 2 criteria) or frail (3–5 criteria) (Fried et al., 2001).

### 2.3 Velocity-based resistance training program

The intervention will consist of a VBRT program performed in the bilateral leg press exercise (Selection MD, Technogym, Italy). The exercise sessions will be conducted twice a week (interspersed by at least 48 h) during a total of 9 months (note: 29 active weeks after excluding assessment weeks and vacation breaks). The leg press exercise has been previously demonstrated to be an effective and safe strategy for improving muscle function in frail, institutionalized and hospitalized older adults (Cadore et al., 2014a; Losa-Reyna et al., 2019; Ferrara et al., 2025). Each session will begin with a ∼10-min warm-up consisting of a 4-min walk at usual gait speed and dynamic balance exercises (zig-zag walking, stepping over obstacles, lateral displacements, and changes of direction). For the main part of the exercise session, the participants will perform either 4 sets (for those allocated in to the 10% VL group) or 3 sets (for those allocated into the 20% VL group) at 60% of F_0_ in the leg press exercise (except for the first week, for which the intensity will be set at 50% of F_0_). The number of sets for each VL group was selected after pilot testing so both groups will perform a similar total number of repetitions in each exercise session. The load corresponding to 60% of F_0_ will be adjusted individually for each exercise session by the monitoring of repetition velocity (Chronojump, Bosco System, Spain) (González-Badillo et al., 2022). Therefore, the absolute load will be decreased when the participants exhibit a mean velocity in the fastest repetition below the target velocity for 60% of F_0_, and the load will be increased when mean velocity in the fastest repetition is higher than the target velocity for 60% of F_0_. The fastest repetition in each set will be used as the reference velocity for controlling intensity and VL, according to previous studies using VBRT (García-Ramos et al., 2021; Sousa et al., 2025). A threshold based on the standard error of measurement (SEM) of mean velocity at 60%F_0_ obtained from our sample in a pilot reliability study (SEM = 0.023 ± 0.004 m·s^-1^) will be considered for increasing or decreasing the load until reaching the target velocity. Similarly, movement velocity will be monitored during the set, and the participants will complete consecutive repetitions until reaching the target intra-set fatigue (i.e., VL threshold) (Sánchez-Medina and González-Badillo, 2011). The participants will be encouraged to perform the concentric phase of each repetition as fast as possible. The eccentric phase will be completed at a 2–3-s pace in order to ensure the reliability of the concentric velocity measurements and to control the physiological adaptations associated with different eccentric durations (Handford et al., 2022). A brief pause (∼1 s) will be imposed between the eccentric and concentric phases. A 3-min rest will be allowed between sets. All sessions will be performed at the same time of the day for each subject (±2 h) and under similar environmental conditions, and will be supervised by qualified strength and conditioning coaches (MSc in Sport Sciences). Attendance will be recorded throughout the intervention.

### 2.4 Follow-up

After the 9-month intervention period, participants only will receive their usual care for a 6-month follow-up period. Usual care will include nursing care, physiotherapist assistance, medical attention, and participation in recreational activities with no structured physical exercise (note: the participants will receive usual care also during the exercise intervention period).

### 2.5 Statistical analysis

Data will be presented as mean and standard deviation or 95% confidence interval unless otherwise stated. Normality of distribution will be assessed by the Shapiro–Wilk test. To analyze the effect of VBRT on all outcomes in the whole sample (primary objective), linear mixed-effect models (LMM) will be fitted using Restricted Maximum Likelihood (REML) estimation. The model will include time as a fixed effect and participant ID as a random effect. The control period will be included in the LMM as an additional time level. This will allow for the comparison of change during the non-exercise period against the time-adjusted changes observed during the VBRT intervention. For the secondary analysis, between-group differences after the VBRT (secondary objective I) will be assessed using the same LMM framework and including time and group (10% vs. 20 VL) as fixed effects, and participant ID as a random effect. The residual effects derived from the VBRT program after 6 months of follow-up will be analyzed similarly, with time as a fixed effect and participant ID as a random effect. To preserve Intention-to-Treat (ITT) principles, training adherence will not be included in the primary model. A secondary per-protocol sensitivity analysis using LMM with REML estimation will be conducted across all objectives, including only participants who completed ≥80% of the exercise sessions. Pairwise comparisons will be adjusted using the Bonferroni method. Cohen’s *d* effect sizes will be calculated using the pooled baseline standard deviation and interpreted as: trivial (<0.20), small (0.20–0.49), moderate (0.50–0.79), and large (>0.80). Statistical analyses will be conducted using SPSS (version 26.0, SPSS Inc., United States), and the level of significance will be set at α = 0.05.

## 3 Discussion

This protocol is designed to evaluate the effects of a VBRT program on skeletal muscle size, muscle function, physical performance, disability, cognitive function, and frailty in nursing home residents using a pragmatic and translational approach. In addition, it will compare the effects derived from the use of two different VL thresholds (10% vs. 20% VL), and will examine the residual effects of VBRT after 6 months of exercise cessation.

There are several potential advantages for using a VBRT approach. First, VBRT represents a valid, and most importantly, feasible method to objectively monitor and prescribe RT parameters (Weakley et al., 2021; González-Badillo et al., 2022). This might be especially important for vulnerable frail populations (such as institutionalized older people) to better adjust the RT dose on an exercise session basis. In addition, VBRT relies on performing each repetition as fast as possible, maximizing adaptations in muscle function (Pareja-Blanco et al., 2014), especially for rapid force production and muscle power. Of note, the ability to apply force rapidly (i.e., rate of force development, RFD) and the rate of producing mechanical work (i.e., muscle power) decreases abruptly with aging (Thompson et al., 2014; Alcazar et al., 2021a), and therefore, their optimization is crucial for maintaining mobility and fall prevention at older age (Hester et al., 2021).

Based on the previous evidence on RT intervention in general and frail older populations, we theorize that VBRT will provoke positive adaptations in the investigated outcomes throughout the course of the study in our institutionalized older adults. Nevertheless, different time courses for the different exercise-induced adaptations might be observed throughout the proposed intervention. Short-term benefits (i.e., post 6 weeks) in frailty, muscle function and physical performance are expected among institutionalized or pre-frail older adults (Serra-Rexach et al., 2011; Courel-Ibáñez et al., 2022; Marques et al., 2022; Buendía-Romero et al., 2023; Baltasar-Fernandez et al., 2024). Our research group has previously demonstrated that 6 weeks of RT on the leg press exercise is an effective stimulus to promote muscle function adaptations and improve frailty in community-dwelling frail older adults (Baltasar-Fernandez et al., 2024). In contrast, in line with previous studies, we do not anticipate significant short-term changes in cognitive function (Coelho-junior et al., 2020), disability (Baltasar-Fernandez et al., 2024) or skeletal muscle size (Lixandrão et al., 2016). Additional benefits are expected in physical and muscle function when a longer period of RT is applied. Studies analyzing the time course of physical function and strength adaptations in institutionalized older adults show accumulated improvements (+7%–24%) after extended 10 weeks of exercise training (Courel-Ibáñez et al., 2022). Moreover, longer periods of RT (as proposed in the current study protocol) could lead to significant enhancements in muscle size, disability and cognitive function. Previous research in institutionalized older adults conducting resistance training for 12 weeks has reported improvements in quadriceps femoris ACSA even in nonagenarians (Cadore et al., 2014a). This requirement for a longer duration would be consistent with previous studies analyzing the time-course of muscle mass adaptation in older adults, where changes in vastus lateralis ACSA were observed only after 9 weeks of resistance training (Lixandrão et al., 2016). Regarding cognitive function and disability, previous studies in nursing home residents have reported improvements in MMSE score and Barthel Index after 12 weeks of exercise training (Cancela Carral et al., 2017; Gutiérrez-Reguero et al., 2024). Indeed, previous systematic reviews propose that interventions lasting longer than 16 weeks are required to maximize the effects of RT as an important moderator of cognitive improvements in older adults (Coelho-junior et al., 2020). Therefore, the duration of this protocol with a 9-month intervention period could be crucial to obtaining meaningful hypertrophy and cognitive benefits, as well as noteworthy disability reductions.

Regarding the comparison of different VL thresholds in our VBRT program (secondary objective), both intra-set fatigues (i.e., 10% and 20%VL) have been proven effective in improving physical performance and muscle strength in older adults from residential care facilities or day centers (Marques et al., 2024). This is the only study to compare different VL exclusively among institutionalized older adults. In line with the expected findings, we anticipate no significant differences between the 10%VL and 20%VL groups in physical and muscle function, frailty and disability over the experimental period. Given that this between-group comparison is a secondary objective and the study is not formally powered to detect differences between VL thresholds, this analysis should be interpreted as exploratory and hypothesis-generating, with the resulting effect sizes serving to inform sample size calculations for future adequately powered trials. Although higher VL thresholds (i.e., 40%VL) are associated with greater hypertrophy in young populations (Pareja-Blanco et al., 2020), lower VL thresholds (i.e., 10–20%VL) may generate less fatigue and be sufficient to elicit structural adaptations in older adults living in nursing homes, characterized by low muscle mass (Kimyagarov et al., 2012), with comparable results between groups. If these hypotheses are confirmed, VBRT with 10% VL would be an effective and efficient approach for improving health parameters while imposing lower fatigue (Marques et al., 2022).

In terms of the residual benefits persisting 6 months after exercise cessation (secondary objective), a recent meta-analysis confirms the residual effects of exercise on physical performance in older adults (Buendía-Romero et al., 2025), however, the evidence with institutionalized older adults is scarce, and the residual effect of VBRT remains unexplored. We hypothesize that a deconditioning will occur in skeletal muscle size, muscle function, physical performance, disability, cognitive function, and frailty after 6 months of usual care without regular exercise in older adults, as supported by previous studies in non-institutionalized older adults (Hakkinen et al., 2000; Zech et al., 2012; Coetsee and Terblanche, 2015; Grgic, 2022). However, despite the predictable decline in several outcomes from the levels achieved immediately after the VBRT intervention, these may remain significantly elevated compared to baseline after several months of exercise cessation (Baltasar-Fernandez et al., 2024). Notably, a rapid decline is common in institutionalized older adults (Serra-Rexach et al., 2011; Telenius et al., 2015; Caldo-Silva et al., 2021), so the long-lasting benefits could be conditioned by the individual exercise response and the age of the participants recruited in this study (Toraman, 2005; Buendía-Romero et al., 2025). Despite the advanced age of institutionalized older adults, the large benefits expected from the VBRT intervention are expected that contribute to significant residual effects after 6 months of usual care. This follow-up is crucial for understanding whether VBRT can induce benefits not only during a prolonged training period but also residual effects.

Due to the pragmatic and translational nature of this study, its results will be highly relevant for both nursing home staff and residents. If the long-term adherence and benefits derived from VBRT are proven in institutionalized older adults, device-based approaches in RT could be a strategy to optimize exercise-related health benefits. Additionally, the findings would support the implementation of innovative actions to promote exercise practice in people living in nursing homes.

## Data Availability

The original contributions presented in the study are included in the article/Supplementary Material, further inquiries can be directed to the corresponding author.
